# Obstructive lower urinary tract symptoms (LUTS) as the initial presentation of penile paraffinoma: a case report and literature review

**DOI:** 10.11604/pamj.2021.38.265.28084

**Published:** 2021-03-15

**Authors:** Asterios Symeonidis, Evangelos N. Symeonidis, Chrysovalantis Toutziaris, Georgios Dimitriadis

**Affiliations:** 1 Department of Urology, 424 General Military Hospital of Thessaloniki, Thessaloniki, Greece,; 2 First Department of Urology, Aristotle University of Thessaloniki, School of Medicine, G. Gennimatas General Hospital, Thessaloniki, Greece

**Keywords:** Penile paraffinoma, lipogranuloma, paraffin oil, phimosis, LUTS, case report

## Abstract

Penile paraffinoma is a rare condition after penile injection of liquid paraffin or other mineral oils, with well-documented debilitating complications. Nevertheless, such injections are still performed by people of Eastern European and Asian descent for cosmetic penile augmentation. We report a case of penile paraffinoma in an otherwise healthy, 30-year-old male, with obstructive lower urinary tract symptoms (LUTS) as the sole complaint at presentation in the emergency department and a conservative approach. This case report describes an unusual presentation of penile paraffinoma in a young man and aims to raise public and physician awareness regarding disease manifestation to prevent high morbidity from delayed diagnosis and treatment.

## Introduction

The positive correlation of penile size with physical strength and virility has been a universally accepted postulate throughout the entire history of mankind [1, 2]. The injection of high-viscosity fluids, such as paraffin oil, paraffin balm, mineral oils, silicone, petroleum jelly, cod liver oil, and nandrolone decanoate [2-4], for the remodeling and augmentation of penile contour, has been described in some primitive tribes [1] and the ancient Indian text of Kama Sutra [4]. It was popularized in the early 1900s with hard and soft paraffin and its destructive complications [5] and still reported in Eastern Europe, Russia, and Asia, with significant case series coming mainly from Korea in the 1990s [1, 6, 7]. Penile paraffinoma, also known as sclerosing lipogranuloma or oleoma, is an unusual but well-documented sequela after these injections [8]. Due to the lack of the necessary enzymes, the human body cannot assimilate exogenous oils, leading to mass formation [4]. Voiding dysfunction represents an uncommon initial manifestation and usually is not the sole symptom on presentation. We report a case of penile paraffinoma in a 30-year-old male presenting with obstructive lower urinary tract symptoms (LUTS) alone, and a brief review on this entity (Annex 1).

## Patient and observation

A 30-year-old uncircumcised, white, Bulgarian construction worker presented to our emergency department with a 48-hour difficulty in emptying the bladder, not continuous urine stream, and dribbling of urine. During the triage process, he denied trauma or force of any kind to his external genitalia. His past medical and surgical history was otherwise unremarkable, and he did not take any medications. He did not smoke, nor did he consume alcohol. Upon further questioning by the urology resident, he reluctantly admitted to having performed a series of three single shots of paraffin oil self-injections over three weeks period for penile girth augmentation while living in his home country, Bulgaria, four years ago. Lastly, the patient reported a fair number and quality of erections throughout the day, but the visual feeling of the presence of the penile aesthetic deformity was what made him abstain from sexual intercourse.

**Figure 1:**
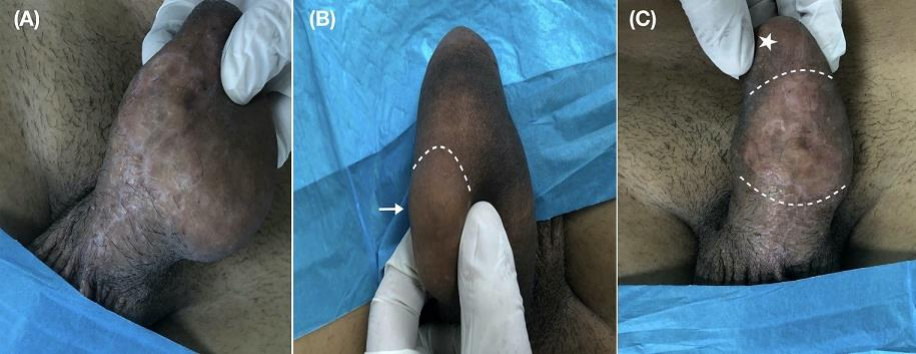
A) indurated mass on the ventral penile shaft; B) paraffinoma mass extending laterally and dorsally (white arrow, dotted lines delineating the lesion borders); C) paraffinoma mass on the ventral penile shaft with associated thinning of the epidermis (lesion between the white dotted lines, white asterisk highlights phimosis)

**Figure 2:**
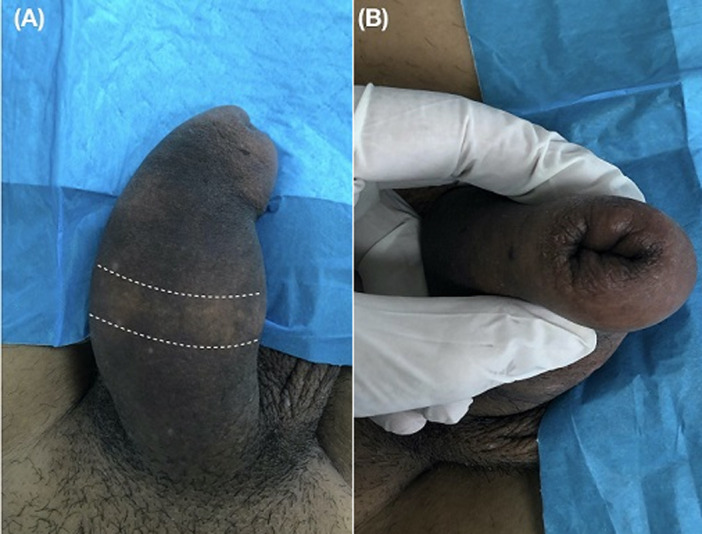
A) multiple injection sites appearing as several white skin spots and a linear area of depigmentation on the dorsal penile shaft (area between the white dotted lines); B) phimosis, foreskin view at maximum retraction

On physical examination, an indurate, non-tender mass of approximately 5 cm (dotted line) was palpated on the ventral aspect of the penile shaft, extending laterally to involve the dorsal surface partially (Figure 1). Therefore, a clinical diagnosis of paraffinoma of the penis was made. Skin findings were remarkable for atrophic changes with thinning of the epidermis and loss of hair follicles. A linear area of depigmentation and several white skin spots, designated by the patient as the injection site, was visible on the dorsal penile shaft (Figure 2). The involvement of the glans penis could not be macroscopically assessed as it was hindered by phimosis (Figure 2). No urethral discharge, involvement of inguinal lymph nodes and lower abdominal wall, or ulceration was identified. Full blood count, serum biochemical analysis, urinalysis, and plain radiographs of the chest were normal. Both a magnetic resonance imaging (MRI) of the penis and scrotum to assess the extent of surrounding structures involvement and surgical excision of the penile mass was offered. Still, the patient viewed a conservative approach as more suitable for his needs. A follow-up visit at one week was scheduled, and the patient was discharged. The patient never appeared for his appointment and was lost to follow-up.

## Discussion

The terms paraffinoma, sclerosing lipogranuloma, and oleoma are used interchangeably in the literature to describe the tissue response´s pathology to mineral oils [9]. Often, the condition is defined by the injected material, hence called paraffinoma or vaselinoma [10]. The discovery of paraffin from beechwood tar from Reicherbach can be traced back to 1830 [11]. Its first cosmetic use came approximately 70 years later, in 1899, by Robert Gersunny, who used solid paraffin injections into the scrotum for testicular replacement after bilateral orchiectomy in a young man who suffered from genital tuberculosis [7], and into the bladder for treating urinary incontinence [11]. Gersunny´s initial promising results paved the way for broader use of oil injections in cosmetic medicine in the first half of the 20^th^ century [12], even though the deleterious effects were identified as early as 1906 by Heidingsfeld [4]. Examples include the correction of facial wrinkles [12], cleft palate [12], baldness [4], and augmentation of muscle, penis, and breast [4], especially in women and male to female transgender individuals [9]. This practice´s scope expanded to include non-cosmetic applications, such as treating hemorrhoids and inguinal hernias [7], repair of urinary fistulas [12], and symptomatic treatment of premature ejaculation and erectile dysfunction [11].

Nowadays, paraffin or injections of other oil types are still performed globally [2] by non-medical personnel or self-injected [13], almost invariably for penile augmentation. The desire for augmentation may stem from the need to boost sexual performance, enhance sexual satisfaction of the partner, or treat erectile dysfunction [1, 5, 13]. In a report of 25 men, all of whom were prisoners and beggars with genital tattoos, Pehlivanov *et al*. have proposed bravery, imitation among inmates, and self-destructive behavior in the setting of a distressing environment as potential motives [1]. A study of Myanmar fishers in Thailand also associated penile oil self-injections with risky sexual behavior, namely engaging in commercial sexual activity and lower use of condoms [4]. The latency period between injection and onset of complications can vary from a couple of days to a maximum of 40 years, as recorded in a case by Eandi *et al*. [4, 8], with a mean time of 1-2 years [5, 8].

Nevertheless, the presentation time may differ as many patients delay a doctor´s consultation when a sensitive topic is at hand. This translates into various signs and symptoms on presentation, including penile deformity with palpable, subcutaneous, indurated masses [4], phimosis, inflammation, ulceration [2, 9] and/or necrosis as a result of infection or mass pressure [11]. Painful erections due to the paraffinoma´s pressure during erection [11] or erectile dysfunction due to skin fibrosis and difficulty in vaginal intercourse [6, 9] may also be present. There has been a handful of case series reporting on voiding dysfunction. In the largest series of complications following penile self-injections, Svensøy *et al*. reported voiding complaints to only 28 out of 680 patients studied (4.1%), with penile pain being the most common symptom in 571 out of 680 patients (84%) [4]. To the best of our knowledge, there have been no previous reports of obstructive LUTS on a man aged 30 years old, as a single complaint on presentation, without penile pain or painful erections. Our case bears a close resemblance to De Siati *et al*.´s case report, in terms of patient´s age, 30 and 27 years old respectively, and delayed presentation, approximately five years after the time of injections. In contrast to our report, their patient was the first case of acute urinary retention and severe penile pain [14]. A case series by Manny *et al*. also reports on three patients, aged 39 to 47, with voiding dysfunction, yet their chief complaint was pain during erections or scrotal pain [3]. Similar to the mechanism proposed by Svensøy *et al*. we speculate that the skin color change in our patient was the result of skin atrophy [4].

Additionally, the local migration of paraffin seen in our case, from the dorsal injection site laterally and ventrally, is in agreement with previous authors. Paraffin may also invade the regional lymph nodes mimicking neoplasia or inguinal hernia, anterior abdominal wall, spermatic cords, and corpora cavernosa at a later stage [3, 5, 7, 8, 11]. Complications following local migration include paraffin embolism, organ infarction, and even death following pulmonary dissemination [2, 11]. Lastly, squamous cell carcinoma linked to mineral oil injection 35 years prior to presentation has also been recorded by Ciancio *et al*. [9]. Ultrasonography and MRI may be valuable in assessing the extent of the inflammation and the aforementioned structures´ involvement. Thus, their role is crucial in operative planning, as complete excision of the granuloma necessitates a preserved and unaffected Buck´s fascia [8, 9]. In our case, paraffinoma diagnosis was in congruence with the patient´s history and physical exam. Hence, we agree with Rosenberg *et al*. that in such cases, the histologic examination may not be required [6]. While admitting the use of injections appears to be the most critical factor in making the diagnosis, in the majority of cases, patients are reluctant to do so [1, 4, 9, 12]. In that regard, chemical analysis of the injected material may be needed, as opposed to our patient who admitted performing injections and was aware of the material used [3, 5]. The presence of injected foreign material can also be confirmed in histopathological examination [9], along with a granulomatous chronic inflammatory reaction surrounding areas of coalescing fat droplets in the subcutaneous fat, which is described as “Swiss cheese appearance” [2, 5, 8, 9].

Owing to paraffin´s innate ability to resist breakdown and tendency to recur if incompletely excised [6, 12], the mainstay of treatment of penile paraffinoma is complete excision of the foreign material, affected skin, and its subcutaneous layer followed by reconstruction of the skin defect [4, 7, 13]. Steffens *et al*. advise against the excision of subcutaneous tissue only, as necrosis of the epidermis may ensue due to decreased blood supply [7]. Nyirády *et al*. argue that preservation of the epidermis may be an option in acute episodes, less than 14 days after injection, when the subdermal layer and blood flow are most likely unaffected. Accordingly, they advocate a surgical treatment in the acute phase for the best aesthetic and functional results [10]. Following complete excision primary closure, scrotal skin flap, Cecil´s scrotal implantation, and split thickness skin graft (STSG) are among the procedures employed for penoplasty. These are lengthy, complex reconstruction procedures that occasionally do not yield the desired outcome [6, 11]. In a series of 19 patients by Lee *et al*. 17 (89.45%) were treated successfully, using a scrotal skin flap supplied by the posterior scrotal branch of the internal pudendal artery with the added advantage of hairlessness [5]. Shin *et al*. introduced the inverted V-shape anastomosis instead of the T-style anastomosis, between the ventral coronal skin and scrotal flap, to deal with complications at the ventral anastomosis site, like necrosis, wound dehiscence and delayed healing. However, 2 of 14 patients in the new technique group still complained about mild shortening of penis and traction during erection [13]. To address the issue of penile length shortening, Sun Wook Kim *et al*. performed a Y-V incision on the pubic symphysis in addition to the bipedicled scrotal flap [11].

Nevertheless, conservative management consisting of antibiotics, oral corticosteroids, painkillers, or watchful waiting for patients who do not opt for surgery, such as in our case, has also been reported [2, 4, 8]. In their series, Svensøy *et al*. treated 637(93.7%) patients with antibiotics irrespective of treatment (surgical or conservative), proclaiming their use as mandatory for secondary infections and surgical prophylaxis [4]. Rosenberg *et al*. recommended a nonsurgical approach for patients who want to maintain the penile enlargement, are scared of the surgery, or face language and cultural barriers in communication that jeopardize obtaining informed consent and following-up with them [6].

## Conclusion

Penile paraffinoma represents an uncommon yet re-emerging condition in some places where penile oil injections for penile augmentation are prevalent or a new reality in others due to the global shifts in populations. Together with a thorough history and clinical examination, high clinical suspicion is required when a young man presents with obstructive LUTS. Raising public and physician awareness about the debilitating complications and clinical manifestations, respectively, is crucial for prevention, early diagnosis, and treatment.

## Annex

**Annex 1**: studies reporting on penile paraffinoma cases
